# The role of macrophages in liver metastasis: mechanisms and therapeutic prospects

**DOI:** 10.3389/fimmu.2025.1542197

**Published:** 2025-02-17

**Authors:** Qin Yuan, Linlin Jia, Jiahua Yang, Wei Li

**Affiliations:** Putuo Hospital, Shanghai University of Traditional Chinese Medicine, Shanghai, China

**Keywords:** macrophages, tumor-associated macrophages, liver metastases, tumor microenvironment, traditional Chinese medicine, therapy

## Abstract

Metastasis is a hallmark of advanced cancer, and the liver is a common site for secondary metastasis of many tumor cells, including colorectal, pancreatic, gastric, and prostate cancers. Macrophages in the tumor microenvironment (TME) promote tumor cell metastasis through various mechanisms, including angiogenesis and immunosuppression, and play a unique role in the development of liver metastasis. Macrophages are affected by a variety of factors. Under conditions of hypoxia and increased acidity in the TME, more factors are now found to promote the polarization of macrophages to the M2 type, including exosomes and amino acids. M2-type macrophages promote tumor cell angiogenesis through a variety of mechanisms, including the secretion of factors such as VEGF, IL-1β, and TGF-β1. M2-type macrophages are subjected to multiple regulatory mechanisms. They also interact with various cells within the tumor microenvironment to co-regulate certain conditions, including the creation of an immunosuppressive microenvironment. This interaction promotes tumor cell metastasis, drug resistance, and immune escape. Based on the advent of single-cell sequencing technology, further insights into macrophage subpopulations in the tumor microenvironment may help in exploring new therapeutic targets in the future. In this paper, we will focus on how macrophages affect the TME, how tumor cells and macrophages as well as other immune cells interact with each other, and further investigate the mechanisms involved in liver metastasis of tumor cells and their potential as therapeutic targets.

## 1 Introduction

**Figure 1:**
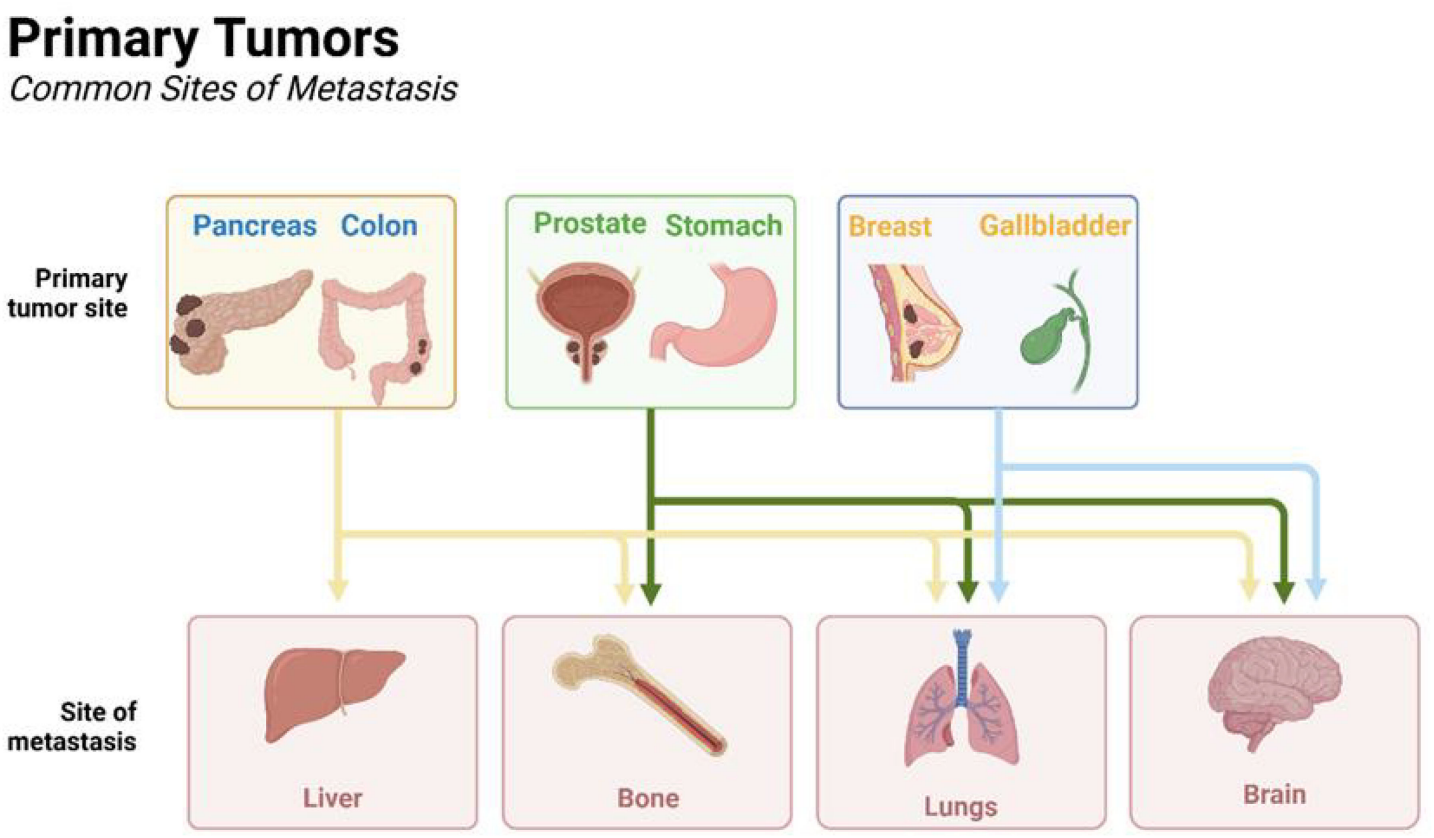
Primary and metastatic sites of different tumor cells. The liver serves as a common site of metastasis for tumor cells, for example pancreatic and colorectal cancers are more common. Created with BioRender.com.

Metastasis is the leading cause of cancer-related death, and the liver is the most common site of cancer metastasis. Liver metastases are highly invasive and refractory. They are a major cause of cancer morbidity and mortality (1). As shown in **Figure 1**, primary tumors of different tissues and organs can metastasize to different sites, and liver metastases occur at advanced stages in a variety of tumor types, including colorectal cancer (CRC), pancreatic cancer (PDAC), breast cancer, gallbladder cancer (GBC), gastric cancer (GC), endometrial mesenchymal sarcoma (ESS), and lung adenocarcinoma (LUAD). However, liver metastases still only benefit from immunotherapy in a small number of patients despite the emergence of emerging therapeutic techniques such as immunotherapy, which may be due to macrophage-mediated factors and affects the patients’ prognosis. Macrophages are a kind of immune cells of the organism, accounting for a large proportion in the tumor microenvironment, and macrophages in the tumor microenvironment are inextricably linked to the emergence of liver metastasis of tumor cells. Tumor cells secrete a variety of factors, such as cytokines and vascular endothelial generating factor, to recruit macrophages and promote macrophages to secrete certain factors to promote the formation of the pre-metastatic ecological niche (2). In the tumor microenvironment, macrophages interact with tumor cells and other immune cells to form an immunosuppressive microenvironment that promotes tumor cell survival and liver metastasis.

Nowadays, emerging therapeutic techniques for liver metastasis are constantly developing. Stereotactic radiation therapy (SBRT) and some immunotherapy are used for the treatment of liver metastasis. However, due to the complexity of the tumor microenvironment, the effectiveness of immunotherapy has been limited. With the emergence of single-cell sequencing technology and in-depth understanding of the molecular mechanisms of cancer occurrence and progression, the development of a more specific and effective therapeutic approach will become the focus of future research. Tumor cell liver metastasis is not a simple disease, and a series of changes occur during the metastatic process. Through in-depth study of the correlation of macrophages in the process of tumor cell liver metastasis and the discovery of new targets, a reference basis is provided for the development of personalized therapeutic methods and new strategies. This provides a broad future for the patient’s quality of life and the patient’s prognosis through the use of new medications and new personalized interventions for treatment.

## 2 Classification of macrophages

**Figure 2:**
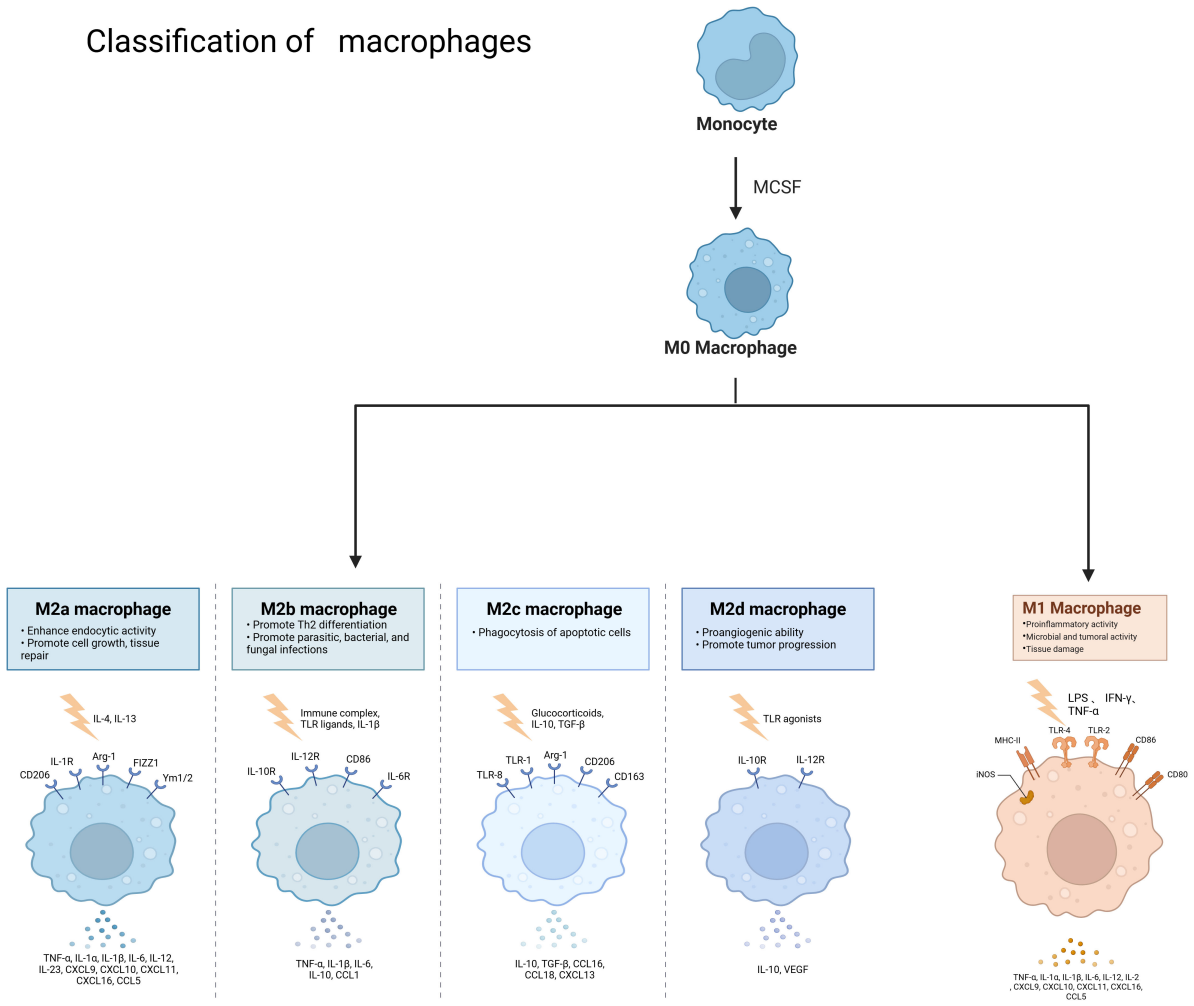
Classification of macrophages and the role of different cell types. Monocytes become M0 macrophages upon M-CSF stimulation, and M0 macrophages can become M2a, M2b, M2c, M2d and M1 types under different conditions, different types of macrophages due to stimulation by different factors and thus have different functions. Created with BioRender.com.

Macrophages are widely distributed innate immune cells. They play different roles in a variety of diseases, such as autoimmune diseases, tumors, and parasitic infections. Macrophages were described by Elie Metchnikoff in 1905. They are found in several parts of the body and have different names depending on their location, such as microglia, Kupffer cells, osteoclasts, bronchoalveolar macrophages, intestinal tissue macrophages, and epidermal Langerhans cells, among others. They have three main sources: macrophages/dendritic cell progenitors (MDPs) derived from prenatal embryonic precursors (yolk sacs or fetal livers) or from bone marrow that give rise to monocytes. They play an irreplaceable role in the maintenance of collective homeostasis (3–5). They have three basic functions: phagocytosis, exogenous antigen presentation, and immunomodulation through cytokine and growth factor secretion (6).Macrophages are highly plastic cells that undergo various forms of functional activation in response to different signals. As shown in **Figure 2**, macrophage classification is usually divided into classically activated macrophages (M1 macrophages) and selectively activated macrophages (M2 macrophages). However, this classification is relatively simplistic. Some studies have mentioned that there are also tumor-associated macrophages (TAMs) and CD169 macrophages. The functions of M1 and M2 macrophages are diametrically opposed. M1-type macrophages have an antitumor effect, while M2-type macrophages promote tumorigenesis and progression. They act on two major lymphocyte subpopulations: Th1 and Th2 cells. M1-type macrophages are mainly activated by interferon (IFN)-γ, tumor necrosis factor (TNF)-α, or lipopolysaccharide (LPS). M2-type macrophages are mainly activated by Th2-associated cytokines (e.g., IL-4, IL-10, and IL-13) and lactate, among others (7). HIF1α is indispensable in the activation induction process (8). M1 macrophages are mainly dependent on aerobic glycolysis, whereas M2 macrophages rely on oxidative metabolism (9). Macrophages can be polarized into M2a, M2b, and M2c subtypes by IL-4, LPS, IL-1β, and IL-10, respectively (10). They contain large amounts of intracellular S100A4, which enhances pre-tumor macrophage polarization by controlling PPAR-γ-dependent fatty acid oxidation induction (11).

### 2.1 Classically activated macrophages (M1 macrophages)

M1 macrophages are typically induced by stimulation with lipopolysaccharide (LPS), interferon-gamma (IFN-γ), tumor necrosis factor-alpha (TNF-α), or granulocyte-macrophage colony-stimulating factor (GM-CSF). They are characterized by high expression of MHC class II molecules, which are involved in antigen presentation, and CD86 molecules, which play a role in T cell co-stimulation. M1 macrophages can secrete TNF-α, interleukin-6 (IL-6), interleukin-1β (IL-1β), IFN-γ, interleukin-12 (IL-12), nitric oxide (NO), and reactive oxygen species (ROS) (12). They promote inflammation and sterilization, block ongoing damage processes, and prevent over-repair (13). Additionally, M1 macrophages have an oncogenic function by releasing large amounts of IL-12, thereby impeding tumor growth (14). The M1 phenotype has been associated with various diseases, including chronic obstructive pulmonary disease (COPD) (15), sepsis (16), diabetic foot ulcers (DFUs) (17), and atherosclerotic lesions (18). M1 macrophages induce TLR4/AP1 signaling in pre-osteoblasts, and their paracrine secretion inhibits osteoclastogenesis, contributing to alveolar bone destruction in periodontitis (19). Exosomes from M1 macrophages transfer miR-628-5p to hepatocellular carcinoma (HCC) cells, inhibiting the expression of human methyltransferase-like 14 (METTL14). This, in turn, suppresses the m6A modification of circFUT8 and the development of HCC (20). Phospholipase D4 (PLD4) has been shown to enhance the anti-tumor effects of M1 macrophages in colon cancer cells (21). M1 macrophages carrying miR-16-5p-derived exosomes inhibit gastric cancer (GC) progression by activating T-cell immune responses through PD-L1 (22). Selective class IIa HDAC inhibitors exert anti-tumor effects on colorectal cancer by promoting M1 macrophage polarization and enhancing the efficacy of PD-1 blockade (23). Macroporous hydrogel (M1LMHA) can secrete various factors, including TNF-α, IFN-γ, and IL-12, and it exerts anti-tumor effects on melanoma by repolarizing M2-type to M1-type macrophages through the NF-κB pathway (24). Chemokine ligand 13 is more highly upregulated in M1 macrophages than in M0 macrophages (25). M1 macrophage-derived exosomes, engineered to promote M1 polarization and target IL4R, inhibit tumor growth by reprogramming tumor-associated macrophages (TAMs) into M1-like macrophages (26). miR-18a inhibits liver metastasis by inducing M1 macrophages (27).

### 2.2 Selectively activated macrophages (M2 macrophages)

#### 2.2.1 M2a

IL-4 and IL-13 are the primary inducers of M2a macrophages. These macrophages secrete TGF-β, IGF, and fibronectin, which facilitate wound healing and fibrosis. M2a macrophages are predominantly found in the renal tubular interstitium, particularly in cases of renal pathologies such as segmental glomerulosclerosis and tubular atrophy/interstitial fibrosis (28). They have also been identified as a significant source of early fibrosis following cerebral ischemia (29). Moreover, M2a macrophages possess bactericidal capabilities (30). CCL22 can polarize cervical cancer-associated tumor-associated macrophages (TAMs) into M2a macrophages (10). However, the elevated expression of chitinase 3-like 1 protein (CHI3L1), a secreted glycoprotein, in M2a macrophages promotes tumor cell invasion and metastasis by upregulating matrix metalloproteinases (MMPs) in various tumor cells. In addition to its role in cancer, CHI3L1 also plays an anti-inflammatory role in several other inflammatory diseases. CHI3L1 secreted by M2a macrophages contributes to the metabolic imbalance of the extracellular matrix in rat intervertebral disc degeneration by activating the IL-13Rα2/MAPK pathway (31).

#### 2.2.2 M2b

M2b macrophages are a subtype of M2 macrophages that have garnered attention due to their potent immunomodulatory and anti-inflammatory effects. Also known as regulatory macrophages, they are activated by immune complexes, lipopolysaccharide (LPS), and toll-like receptor (TLR) ligands. Additionally, it has been shown that activated lymphocyte-derived DNA (ALD-DNA) can also promote macrophage polarization towards the M2b phenotype (32). M2b macrophages are characterized by the expression of markers such as IL-6, tumor necrosis factor-alpha (TNF-α), CD86, TNFSF14, IL-10, CCL1, and SPHK1 (33). These macrophages secrete factors like IL-10, IL-6, and IL-1, which can exert pro-tumorigenic effects. Notably, IL-10 possesses strong anti-inflammatory properties. M2b macrophage exosomes mediate the response to glucagon by modulating the CCL1/CCR8 axis. This exocytosis also exerts a protective effect against dextran sulfate sodium (DSS)-induced colitis through the same CCL1/CCR8 axis (34). Furthermore, alcohol and LPS have been found to induce macrophage M2b polarization by activating the MAPK/P38, MAPK/ERK, and NF-κB signaling pathways. This activation leads to an increased expression of inflammatory cytokines such as TNF, IL-1β, and IL-10 (35).

#### 2.2.3 M2c

M2c macrophages are characterized by their role in tissue remodeling and regeneration (36). They also perform several other functions, including angiogenesis, matrix maturation, and phagocytosis (37). M2c macrophages are primarily activated by glucocorticoids or IL-10. They promote the epithelial-mesenchymal transition (EMT) of human renal proximal tubular epithelial cells (HK-2) (38). M2c macrophages secrete TGF-β and IL-10, and they are involved in phagocytosis, immunosuppression, angiogenesis, and the development of tissue fibrosis.

#### 2.2.4 M2d

**Figure 3:**
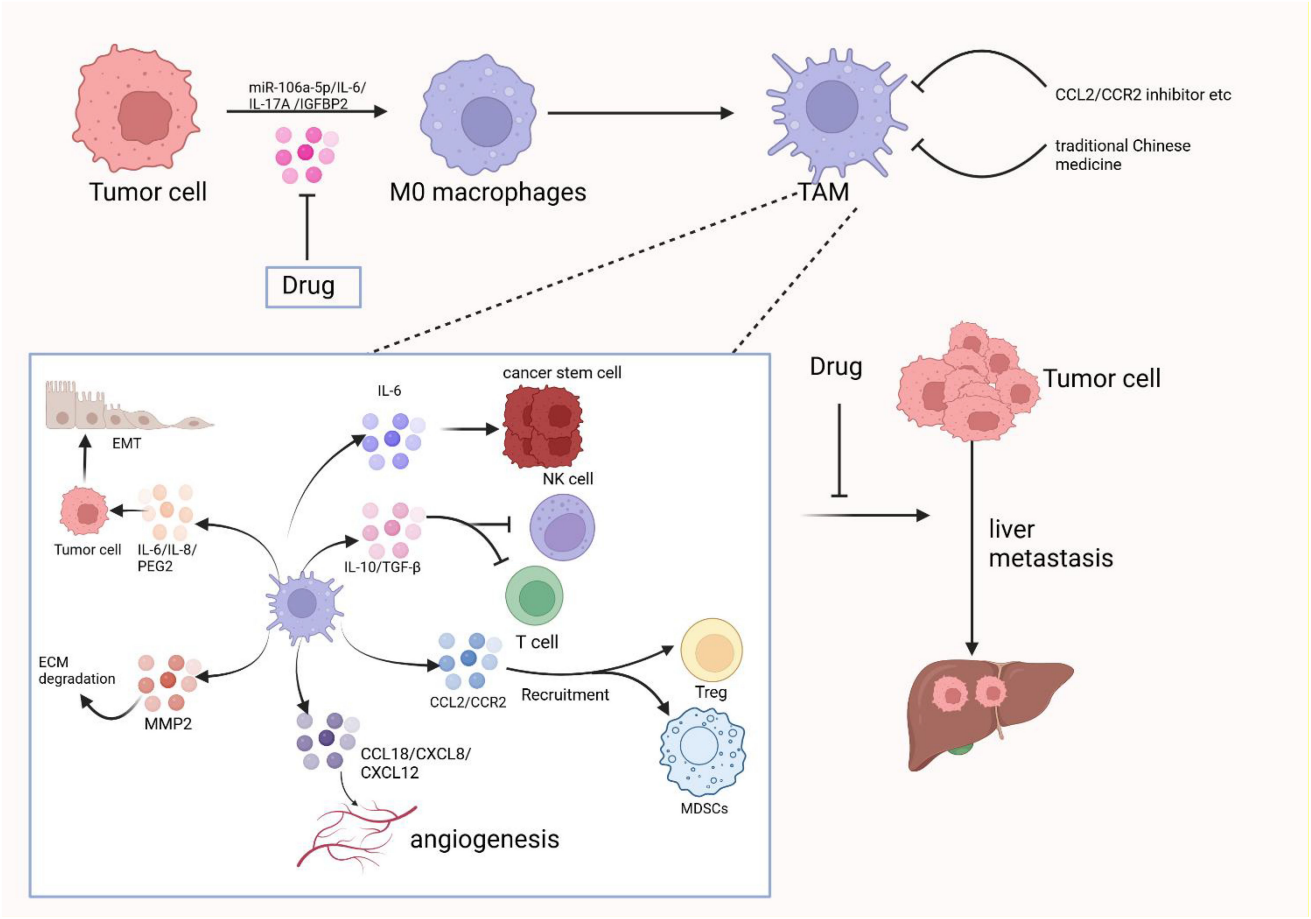
The crucial role of tumor-associated macrophages in promoting the progression of liver metastases in the tumor microenvironment and therapeutic strategies to target them. Created with BioRender.com.

M2d macrophages, also known as tumor-associated macrophages (TAMs), are primarily activated by adenosine or IL-6. These macrophages secrete IL-10, TGF-β, and VEGF, which promote angiogenesis and cancer metastasis. They are recruited from circulating monocytes into tumors and are influenced by the presence of cancer to facilitate tumor malignancy and progression. M2d macrophages are often referred to as tumor-associated macrophages (TAMs) (5). In the tumor microenvironment, various secreted factors and exosomes from tumor cells can promote macrophage polarization towards the M2 type. For instance, circPOLQ can enhance M2 macrophage polarization via CRC cell-derived exosomes (39). Similarly, the cancer-associated fibroblast (CAF)-related gene insulin-like growth factor-binding protein 2 (IGFBP2) plays a role in this process, as mentioned in gliomas (40). The exosome miR-106a-5p from colorectal cancer is also involved in M2-type polarization (41). In cervical cancer, IL-17A has been found to promote M2 macrophage polarization, thereby facilitating metastasis. Additionally, lactate secreted by cervical cancer promotes M2-type polarization by upregulating GPD2 through histone lactylation (42, 43). In lung cancer, tumor-derived γ-aminobutyric acid (GABA) promotes macrophage M2 polarization by activating the JAK1/STAT6 pathway and inhibits M1 polarization by suppressing the JAK2/STAT3 pathway (44). Nowadays, M2 macrophage polarization is not only mediated by IL-6 and other factors, but an increasing number of exosomes, secreted factors, and even amino acids have been discovered to play a role. TAMs can promote tumor progression, metastasis, and treatment resistance. Macrophages infiltrating tumor tissues are driven by tumor-derived and T-cell-derived cytokines (45). TAMs, as a specific phenotype of M2-like macrophages, are phagocytes of unknown origin (4). They typically exist in an M2-like phenotype with anti-inflammatory functions and shape the tumor microenvironment by secreting various immunosuppressive cytokines and modulating cytotoxic T lymphocyte (CTL) activity (46). Hypoxia, a hallmark of the tumor microenvironment, can promote macrophage polarization into TAMs to a greater extent (47). TAMs are the most abundant cell type in the tumor microenvironment (TME) and are mainly involved in angiogenesis, immunosuppression, tumor cell invasion, stromal remodeling, and tumor resistance to chemotherapy. They also promote immune escape of tumor cells (48). TAMs can promote tumor cell initiation and metastasis, inhibit T cell-mediated anti-tumor immune responses, and stimulate tumor angiogenesis and subsequent tumor progression (49). They contribute to the hematogenous spread of cancer cells, especially liver metastasis (50). Increased TAM recruitment and M2 polarization are observed in liver metastatic lesions compared to primary sites of human CRC tissue (51). Kupffer cells enhance metastasis formation in the liver (52) and also induce hepatic metastasis formation through AT1a signaling by inducing TGF-β1 (53). Fatty Acid Binding Protein 4 (FABP4) in TAMs may contribute to hepatic metastasis formation by inactivating the NF-κB-IL1α pathway through ubiquitination of ATPB, thereby promoting the proliferation and migration of neuroblastoma cells (NB) (54). STAT1, STAT3, STAT5, and STAT6 have all been associated with TAM function. The release of sphingosine 1-phosphate (S1P) from apoptotic tumor cells stimulates lipid-cotransporting protein 2 (LCN2) production in TAMs and is associated with metastasis (55). Lung cancer particles (L-MPs) induce the release of a key pro-inflammatory cytokine, IL-1β, from M2-like macrophages, which promotes lung cancer development (56). Interstitial flow in the tumor environment, a mechanical stimulus, promotes M2 polarization of macrophages. These polarized macrophages are recruited to the tumor mass, thereby promoting cancer cell invasion and tumor progression (57). Co-culture of TAMs with tumor-associated neutrophils (TANs) results in altered expression of cytokines and chemokines, producing interleukin (IL-11) and oncostatin M (OSM). These factors activate STAT3 signaling and promote intrahepatic cholangiocarcinoma (ICC) cell proliferation, invasion, and colony formation (58). Collagen Triple Helix Repeat Sequence 1 (CTHRC1) in primary CRC promotes CRC liver metastasis by remodeling infiltrating macrophages through TGF-β signaling (59). Depletion of mitogen-activated protein kinase 4 (MAPK4) in gastric cancer cells is associated with macrophage polarization to tumor-associated macrophages (TAMs). This induces the secretion of macrophage migration inhibitory factor (MIF), thereby promoting macrophage polarization. TAMs activate the epithelial-mesenchymal transition of gastric cancer cells and inhibit the expression of MAPK4, creating a positive feedback loop that induces the secretion of MIF and contributes to TAM polarization. This process can promote gastric cancer liver metastasis (60). CC-chemokine ligand 20 (CCL20) expression is abnormally elevated in TAMs of pancreatic cancer tissues. CC-chemokine receptor 6 (CCR6) acts as a receptor for CCL20 in pancreatic cancer cells, mediating the promotion of pancreatic cancer growth and liver metastasis by CCL20 in a mouse model (61). CRC with liver metastasis has an elevated proportion of infiltrating M2 macrophages compared to CRC without liver metastasis. A positive feedback loop exists in which CRC promotes macrophage M2 polarization through the activation of the JAK2/STAT3 pathway by releasing the exosome miR-106a-5p and inhibiting SOCS6. This, in turn, promotes the occurrence of liver metastasis in CRC (41). Macrophages in the TME microenvironment have a strong lipid uptake capacity. Tumor cell-derived fatty acids, such as monounsaturated long-chain fatty acids (LCFAs), play a role in inducing macrophage polarization to the M2 type. This process is mainly mediated by upregulated CD36 in tumor-infiltrating macrophages, which play a pro-tumorigenic role after phagocytosis of lipids in the tumor cells via a CD36-dependent mechanism (62). As shown in **Figure 3**, in the tumor microenvironment, tumor-associated macrophages play a crucial role in promoting the progression of liver metastasis,therefore, the occurrence of liver metastasis may be suppressed to some extent by inhibiting TAM.

### 2.3 CD169 macrophages

CD169 macrophages are a subpopulation of macrophages primarily found in the lymph node peritoneal sinusoids and the splenic border (10). However, a few are also present in the intestine, liver, and bone marrow. The density of CD169-positive macrophages in lymph node (LN) sinusoids is positively correlated with the density of infiltrating T cells and NK cells in tumor tissues. This suggests that CD169-positive macrophages play a significant role in the anti-tumor immune response of patients with tumors (63). Depletion of CD169 macrophages in tumor-draining LNs leads to increased lung metastasis (64). Radiofrequency ablation (RFA) is a therapeutic approach for treating primary or metastatic hepatocellular carcinoma with small foci. RFA induces limited distant effects in hepatocellular carcinoma. However, the transfer of CD169 macrophages effectively improves RFA-induced distant effects and contributes to the regression of liver tumors (65).

### 2.4 TAM subsets based on single-cell sequencing

**Figure 4:**
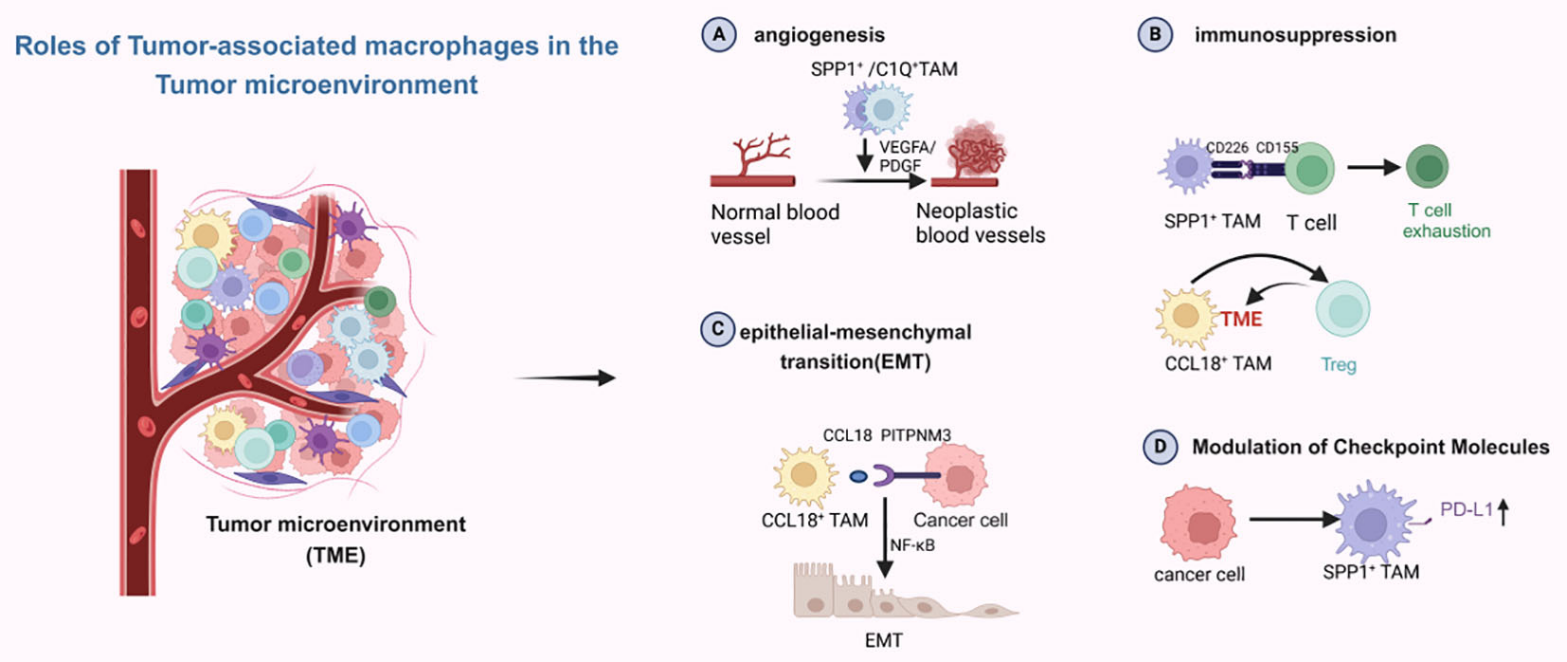
Different subpopulations of macrophages play different roles in the tumor microenvironment. Created with BioRender.com.

With the advent of single-cell sequencing technology, macrophage classification is no longer limited to traditional M subtypes. In the tumor microenvironment (TME), macrophages are divided into two main subsets: C1Q^+^ and SPP1^+^ tumor-associated macrophages (TAMs), as well as two secondary subsets: FCN1^+^ and CCL18^+^ TAMs. The SPP1^+^ and C1Q^+^ TAM subsets can be further divided into distinct populations with varying functions (66). As shown in **Figure 4**, in the tumor microenvironment, each of these subsets express different molecular functions, including immunosuppression and promotion of angiogenesis, with SPP1^+^ TAMs being particularly associated with malignancy. They are also linked to liver metastases and promote angiogenesis (67). C1Q^+^ TAMs and CCL18^+^ TAMs mainly exert immunosuppressive effects. The interaction of SPP1^+^ TAMs with fibroblasts establishes an immunosuppressive metastatic niche, fostering the growth of colorectal cancer cells within this niche (68). Liang et al. have reported similar findings: in oral squamous cell carcinoma, TAMs may potentially activate fibroblasts and promote T cell exhaustion through SPP1-CD44 and CD155-CD226 ligand-receptor interactions. This reshapes the metastatic lymph node microenvironment, thereby facilitating the colonization and proliferation of disseminated tumor cells (69). Xiangxiang Liu et al. (70) reported that SPP1^+^ TAMs enhance the invasion and metastasis of colorectal cancer cells and also create an immunosuppressive microenvironment for hepatocellular carcinoma (71). In non-small cell lung cancer, single-cell RNA sequencing has identified SPP1^+^ macrophages, and another immunosuppressive TAM, CCL18+ TAM, has been discovered. CCL18+ TAMs exert immunosuppressive effects by inhibiting the production of inflammatory cytokines through the suppression of oxidative phosphorylation as their primary metabolic mode. At the same time, CCL18^+^ TAMs can promote proliferation, migration, and epithelial-mesenchymal transition (EMT) in oral squamous cell carcinoma (72). Current research on the relationship between CCL18^+^ TAMs and liver metastasis is limited, and future studies will primarily focus on developing treatments targeting CCL18^+^ TAMs. For SPP1^+^ TAMs, they can upregulate PD-L1 expression while promoting TME matrix remodeling to accelerate tumor progression (73). C1Q^+^ TAMs appear to be associated with T cell exhaustion in the tumor microenvironment, facilitating this process by expressing immune checkpoints, interacting with T cell immune checkpoints, and also directly interacting with endothelial cells to promote the neoangiogenesis required for tumor growth (74). The discovery of the interaction between CD3^+^C1Q^+^ TAMs and CD8^+^CCL4^+^ T cells in hepatocellular carcinoma presents a novel therapeutic target for reversing the immunosuppressive microenvironment in this cancer type. FCN1^+^ TAMs are thought to represent an intermediate stage in the maturation of monocytes into tumor macrophages and are associated with angiogenesis (75). These TAM subsets are more specific than traditional M subsets. Different subsets focus on distinct functions, making them more detailed and precise targets for therapeutic research.

## 3 Mechanisms of macrophage in liver metastasis

**Figure 5:**
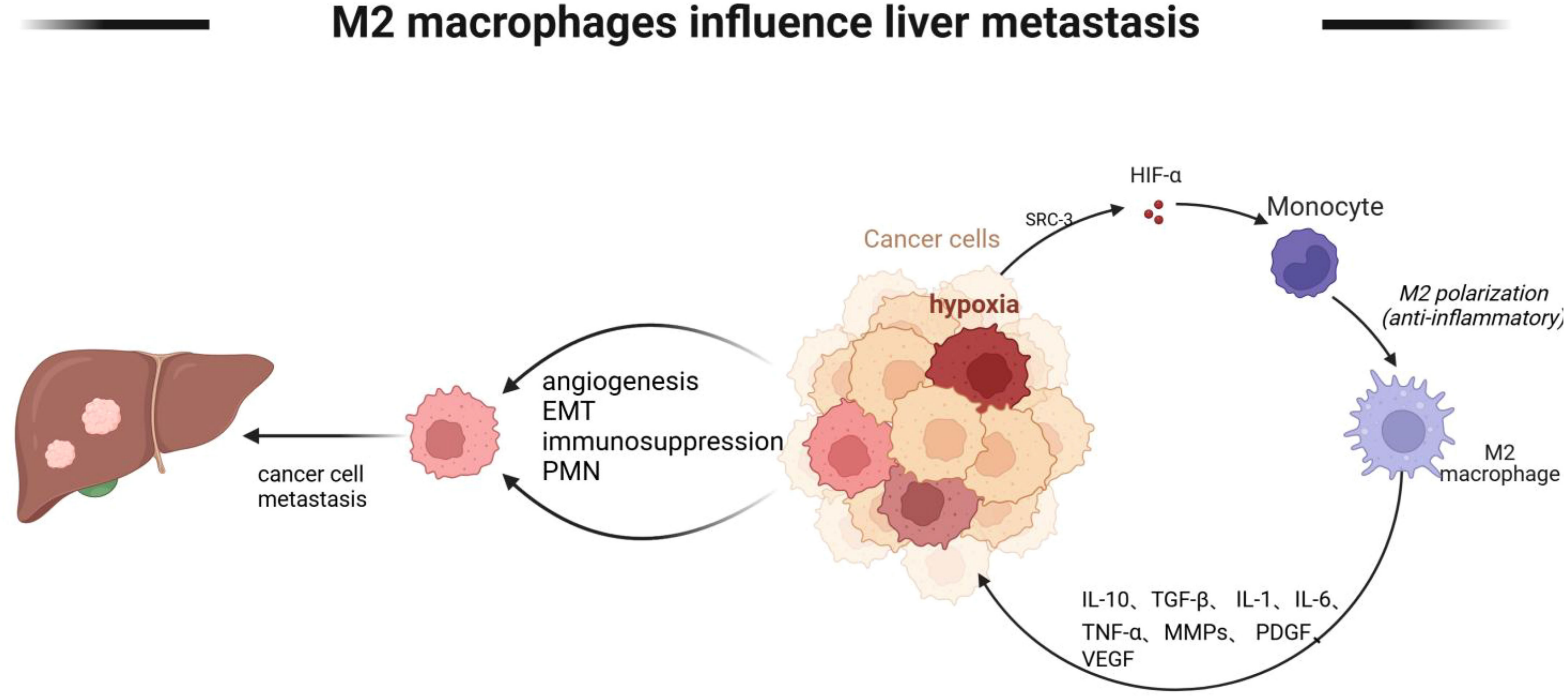
M2 macrophages mediate tumor cell liver metastasis. M2-type macrophages mediate tumor cell liver metastasis Lack of oxygen in the tumor microenvironment promotes monocyte polarization to M2 type, and M2 type macrophages further promote angiogenesis, EMT, and immunosuppressive state of tumor cells through IL-10, TGF-β, and IL-6, and further liver metastasis. Created with BioRender.com.

Tumor cells metastasize through a variety of mechanisms, including angiogenesis, immunosuppression, and establishment of a pre-metastatic ecological niche, etc. Macrophages can mediate these mechanisms. The environment in which tumor cells live is a state of hypoxia, and under hypoxia, macrophages promote the emergence of hepatic metastasis by affecting angiogenesis and immunosuppression of tumor cells, as shown in **Figure 5**.

### 3.1 Angiogenesis

Tumor cells can metastasize through various mechanisms, including increased angiogenesis to facilitate metastasis. Blood vessels are crucial for providing nutrients, maintaining cells, and supporting further growth in new metastases. The tumor microenvironment, where tumor cells reside, is often hypoxic. In this hypoxic state, macrophages are also oxygen-deprived, and HIF-1α stimulates tumor-associated macrophages (TAMs) to produce various factors, such as VEGF, IL-1β, TGF-β1, tumor necrosis factor (TNF)-α, basic fibroblast growth factor (bFGF), and IL-10. These factors induce angiogenesis, thereby promoting tumor cell metastasis (76, 77). CRC cells that overexpress regenerative hepatic protein phosphatase 3 (PRL-3) exhibit increased angiogenesis when co-cultured with TAMs. PRL-3 promotes angiogenesis by activating the NF-κB pathway in CRC cells (78). Additionally, TGF-β and pro-angiogenic growth factor PDGF contribute to solid tumor angiogenesis (79, 80). Studies have shown that M2 macrophage-derived exosomes (MDEs) can promote angiogenesis in mouse aortic endothelial cells (MAECs) *in vitro*. Moreover, M2 MDEs can enhance subcutaneous tumor growth and increase vascular density in mice. Exosomal miR-155-5p and miR-221-5p target E2F2 to promote angiogenesis and growth of pancreatic ductal adenocarcinoma (PDAC) *in vivo* (81). Upregulated miR-25-3p, miR-130b-3p, and miR-425-5p in CRC cells regulate PTEN through activation of the PI3K/Akt signaling pathway, inducing M2 polarization of macrophages. In turn, M2-polarized macrophages promote cancer metastasis by enhancing epithelial-mesenchymal transition (EMT) and secreting vascular endothelial growth factor (VEGF) (82). A similar situation exists in liver metastasis of gastric cancer cells. Hepatic macrophage KC secretes IL-6, hepatocyte growth factor (HGF), VEGF, and matrix metalloproteinases (MMP) 9 and 14. These factors accelerate tumor invasion into the parenchymal and interstitial spaces, promote tumor cell proliferation and angiogenesis, and thus enhance liver metastasis (83).

### 3.2 Immunosuppression

Macrophage-mediated immunosuppression contributes to liver metastasis and a poor prognosis. It can exert its immunosuppressive effects in various ways, such as recruiting cancer-associated fibroblasts (CAFs) and promoting mutual interactions to further create an immunosuppressive environment. Additionally, it induces Treg differentiation, inhibits natural killer (NK) cell activation, and impairs dendritic cell (DC) function (84). Tumor-resident microbiota, such as E. coli, enhances lactic acid production. Lactic acid-cultured Kupffer cells secrete cytokines that promote PD-1 expression in regulatory T cells (Tregs) and reduce CD8 T cell activation (85). In the hypoxic tumor microenvironment, macrophages express PD-L1 in response to HIF-1α secretion. PD-L1 acts as a ligand for the inhibitory receptor programmed cell death protein 1 (PD-1) and suppresses T cell activity, creating a state of immunosuppression. Prostaglandin E2 (PGE2), an immunosuppressant produced by cancer cells and their surrounding macrophages, and high concentrations of PGE2 in the portal vein inhibit liver-associated immunity and promote the formation of hepatic metastases (86). Tumor-associated macrophages (TAMs) may promote gastric cancer (GC) angiogenesis by enhancing VEGF and VEGF-C expression and lymphangiogenesis (87). In colorectal cancer, mucin 1 (ORM1) partially promotes colorectal liver metastasis (CRLM) by inducing macrophage M2 polarization. It also promotes macrophage production of IL-10, which is implicated in regulating the immune response, thereby inducing the formation of an immunosuppressive microenvironment (88). Various complex interactions between myofibroblasts-metastasis-associated fibroblasts (myMAFs) can promote tumor cell metastasis. Macrophage-derived granulin precursors can induce myMAFs through a STAT3-dependent mechanism. Further, myMAF secretion of bone-bridging proteins promotes an immunosuppressive macrophage phenotype, which inhibits cytotoxic T cell function. This, in turn, causes immunosuppression and promotes hepatic metastasis of pancreatic cancer cells (89). Tumor TCF4-induced recruitment and polarization of TAMs may produce an immunosuppressive, pro-metastatic milieu by preventing CD8 T cell infiltration (51).

### 3.3 Establishment of pre-transfer ecological niches

Tumor cells create a susceptible metastatic microenvironment, known as the pre-metastatic niche (PMN), before reaching the metastatic site. In this PMN, CRC exosomes promote the polarization of M2 macrophages. These macrophages, in turn, secrete CXCL12, which increases cancer metastasis through the CXCL12/CXCR4/NF-κB signaling pathway, leading to colorectal cancer liver metastasis (CRLM) (90). Triple-negative breast cancer (TNBC) develops a pre-metastatic niche in the liver during the development of liver metastases. This process is associated with CX3CL1 levels, which promote macrophage migration and breast cancer cell invasion by recruiting CX3CR1-expressing macrophages. Consequently, CX3CL1-CX3CR1 signaling leads to the upregulation of MMP9 (91). The exosome miR-4488 secreted by pancreatic neuroendocrine tumors (pNENs) promotes M2-like polarization by directly targeting and inhibiting RTN3 in macrophages. M2-polarized macrophages then promote pre-metastatic ecological niche formation and advance pNENs liver metastasis by releasing MMP2 (92). CRC-sEVs induce an inflammatory pre-metastatic ecological niche through the miR-21-TLR7-IL-6 axis, playing a key role in promoting liver metastasis (93). Liver metastasis of pancreatic ductal adenocarcinoma (PDAC) has also been linked to the hepatic pre-metastatic niche. Here, exosomal macrophage migration inhibitory factor (MIF) induces the release of transforming growth factor β (TGFβ) from Kupffer cells, which in turn promotes the production of fibronectin (FN) by hepatic stellate cells (hStCs). FN deposits facilitate the colonization of the liver by bone marrow-derived macrophages and neutrophils, resulting in the formation of a pre-metastatic ecological niche (94). Tumor-derived extracellular vesicles (TEVs) enriched with Circ-0034880 promote strong interactions between primary tumor cells and SPP1 ^high^ CD206 tumor-promoting macrophages, fostering PMN formation and CRLM (95).

### 3.4 Protection of circulating tumor cell survival

Tumor cells metastasize through several steps. Initially, they invade the adjacent parenchyma from the confined primary tumor. Subsequently, they enter the circulation and ultimately reach the metastatic site. At this site, they are exposed to certain factors, such as transforming growth factor β (TGFβ) and fibronectin, which enable them to survive.

### 3.5 Epithelial-mesenchymal transition

The process of tumor invasion and metastasis is often associated with the activation of epithelial-mesenchymal transition (EMT), and the link between EMT and tumor-associated macrophages (TAMs) is quite close. The activation of EMT typically requires crosstalk between cancer cells and components of the local tumor microenvironment, including TAMs. TAMs can promote EMT in tumor cells by secreting various factors. For instance, TGF-β and PRL-3 upregulate the expression of CCL26. CCL26 induces TAMs infiltration and increases the expression of IL-6 and IL-8 in tumor cells, which promotes EMT. Fibroblast growth factor 9 (FGF9) may contribute to macrophage recruitment and M2 polarization, and TAMs are involved in regulating the EMT process, further contributing to hepatic metastasis (96). M2-polarized TAMs are further promoted, in part, via TLR4/IL-10 signaling, which enhances EMT in pancreatic cancer cells (97). Additionally, their secretion of CXCL2 promotes EMT in gastrointestinal mesenchymal stromal tumor (GIST) cells (98). In pancreatic cancer, ALOX5 is highly expressed, primarily in macrophages. ALOX5 upregulates the expression of vimentin and downregulates the expression of E-cadherin to promote EMT in pancreatic cancer. It also promotes the M2-like phenotype of human macrophages through the JAK/STAT pathway, and the occurrence of liver metastasis is more pronounced with high ALOX5 expression, as observed in *in vivo* experiments (99).

### 3.6 Induction of autophagy in tumor cells

Autophagy is a crucial protective mechanism that enables tumor cells to withstand harsh living conditions, such as hypoxia and nutrient deprivation. Tumor-associated macrophages (TAMs) can induce autophagy in tumor cells, thereby helping them resist these adverse environments. In particular, TAMs that home around metastatic nests can induce autophagic fluxes in gastric cancer (GC) cells and promote hepatic metastasis through GDNF-GFRA1 signaling. *In vivo* experiments have shown that downregulating GFRA1 significantly inhibits the colonization and formation of metastatic GC ecosystems in the liver. Conversely, this suggests that GFRA1 promotes the formation of hepatic GC metastatic ecosystems. Additionally, it has been found that TAMs act on GFRA1 expressed by GC cells, enhancing their growth ability under poor nutritional conditions through secreted proteins, which may include GDNF from TAMs (100).

### 3.7 Extracellular vesicles and macrophages

In recent years, research on extracellular vesicles (EVs) promoting tumor cell liver metastasis by affecting macrophages has garnered attention. The tumor microenvironment (TME) is a dynamic system where non-tumor cells and cancer cells interact through soluble factors and EVs, releasing a variety of EVs (including exosomes, microvesicles, and many other vesicles) into the environment (101). Within the TME, both tumor cells and macrophages can promote metastasis, drug resistance, and immune escape by secreting microvesicles. The TME often exhibits a hypoxic profile, leading to an increase in exosome production in response to hypoxia-inducible factor (HIF)-1α (102). In colorectal cancer, CRC-EVs activate NOD1 in macrophages, initiating the secretion of inflammatory cytokines and chemokines (IL-6, CCL1, and CCL2) to promote tumor cell migration, while also promoting CRC liver metastasis (CRC-LM) by activating NOD1 (103). Tumor-derived extracellular vesicles (TEVs) can contribute to liver metastasis by fostering the formation of pre-metastatic niches (PMNs), such as small extracellular vesicles (sEVs) enriched with miR-151a-3p (104), and by accelerating GC-LM generation through macrophage polarization and its impact on angiogenesis (105). Tumor cells can also undergo hepatic metastasis via tumor-associated macrophage (TAM)-derived extracellular vesicles (EVs). TAM-EVs can influence tumor cells to undergo epithelial-mesenchymal transition (EMT), characterized by upregulation of N-cadherin and vimentin proteins and downregulation of E-cadherin in CRC cells incubated with TAM-EVs. TAM-EVs primarily affect tumor cells to undergo EMT by upregulating ABCA1 expression in MC-38 and CT-26 cells, decreasing cell membrane and intracellular cholesterol content, and potentially increasing membrane fluidity, which is closely associated with tumor cell metastasis (106). These extracellular vesicles regulate macrophage polarization and differentiation through various mechanisms and signaling pathways, providing a conducive environment for tumor cell survival and growth in the liver (**Table 1**).

**Table 1:** Extracellular vesicles associated with macrophages in liver metastasis.

EV	Mechanism	Reference
sEV-Ezrin (sEV-EZR)	Affects macrophage polarization and promotes liver metastasis	(107)
sEV-miR-106b-5p, sEV-miR-18a-5p, sEV-miR-21-5p, sEV-miR-200a	Promotes the expression of immune checkpoints for PD-L1 in M2 TAMs, such as PD-L1	(108, 109)
EVs-ANXA1	Regulates interactions between TAMs and other cells to induce angiogenesis and matrix degradation	(110)
EV-circ-0034880	Activation of SPP1^high^CD206^+^TAM to remodel TME	(95)
EV-CDC42	Activation macrophages to secrete inflammatory and chemotactic factors promotes metastasis.	(103)
miR-934, miR-122, miR-25, miR-130b. miR-425, miR-203a-3p	Activation of the PI3K/AKT signaling pathway induces M2 macrophage polarization.	(82, 90, 111, 112)
miR-519a-3p	Targeting DUSP2 activates the MAPK/ERK pathway, leading to M2-like polarization of macrophages.	(105)
miR-29a-3p, miR-21-5p	Mediating the interaction between TAMs and T cells creates an immunosuppressive microenvironment.	(113)
miR-21-3p, miR-125 b-5p, miR-181 d-Sp	Promotion of M2-type macrophage polarization by HIF-1αand HIF-2α	(114)
miR-501-3p	Activation of the TGF-β signaling pathway inhibits the tumor suppressor gene TGFBR3.	(115)
miR-6794-5p	Activation of the JAK1/STAT3 pathway induces M2 macrophage polarization.	(116)

### 3.8 Immune checkpoints and macrophages

Immune checkpoints play a crucial role in the immune response process. Previously identified immune checkpoints include PD-1/PD-L1 and cytotoxic T-lymphocyte antigen 4 (CTLA4). By interfering with the binding of immune checkpoints to their ligands, the anti-tumor ability of immune cells can be restored. Nowadays, the study of newly identified immune checkpoints, such as TIM3, LAG3, TIGIT, NKG2D, and CD4, as well as their respective ligands, is on the rise. These checkpoints and ligands are being intensively studied and may become new therapeutic targets in the future (117). The lymphotoxin β-receptor (LTBR) has been identified as a potential immune checkpoint in tumor-associated macrophages (TAMs). Its high expression, duplication, and hypomethylation are associated with a poor prognosis. LTBR maintains the immunosuppressive activity and M2 phenotype of TAMs through the NF-κB and Wnt/β-catenin signaling pathways, making it a promising target for overcoming immune checkpoint resistance (118). In the tumor microenvironment (TME), the expression of PD-L1 and CTLA-4 on TAM surfaces, as well as their ligands, is increased. This is often linked to a poorer clinical prognosis in patients with liver metastatic cancers (6). PD-L1 is upregulated and induces M2 polarization in TAMs (119). The binding of TAM surface PD-L1 and CTLA-4 to the corresponding receptors on T cells can inhibit T cell activation and proliferation, thereby enhancing the immunosuppressive effect (120). Studies have shown that overexpression of PAARH in hepatocellular carcinoma significantly promotes M2 macrophage polarization, increases PD-L1 levels, and enhances cell proliferation, migration, and invasion. It also inhibits M1 macrophage polarization, decreases PD-L2 levels, and suppresses apoptosis (121). TGF-β secreted by M2-TAMs increases glycolysis levels in bladder cancer (BLCA) and inhibits cell proliferation and migration via the pyruvate kinase isoenzyme (122). The pyruvate kinase isoform M2 (PKM2) plays a significant role in PD-L1-mediated immune evasion (122). Macrophages exert immunosuppressive effects in the tumor microenvironment, and their presence can inhibit the action of immune checkpoint inhibitors (123). This may contribute to the poor therapeutic efficacy of immune checkpoint inhibitors in patients with liver metastases and the development of drug resistance. Therefore, targeting macrophages in combination with immune checkpoint inhibitors can synergistically enhance the anti-tumor immune response and help overcome immune resistance.

### 3.9 Signaling pathways and secreted factors associated with macrophage-promoted liver metastasis

Macrophages in the tumor microenvironment promote hepatic metastasis by enhancing immunosuppression and tumor cell proliferation, influencing the formation of the tumor microenvironment, and interacting with tumor cells. Specifically, they facilitate liver metastasis through tumor cell angiogenesis, the establishment of immunosuppression within the tumor microenvironment, and the production of pre-metastatic niches (PMNs), which involve multiple mechanisms and are associated with various secreted factors. Within the tumor microenvironment, macrophages can promote liver metastasis in several ways. For instance, M2-type macrophages can facilitate colorectal liver metastasis (CRLM) by secreting CXCL13, which subsequently activates the CXCL13/CXCR5/NF-κB/p65/miR-934 pathway in colorectal cancer (CRC) cells (111). TGF-β, a pro-fibroblastic cytokine, is secreted by intrahepatic macrophages, which in turn activate hepatic stellate cells (HSCs) and hepatic stromal cells, promoting hepatic PMN formation. Exosomes from tumor-associated macrophages (TAMs) also impact tumor cell liver metastasis. miR-142-5p and miR-202-5p promote pancreatic ductal adenocarcinoma (PDAC) cell invasion and metastasis by inhibiting PTEN-mediated mesenchymal-epithelial transition (MET) (124), and TAMs also suppress tumor suppressor genes to facilitate PDAC cell metastasis. In addition, macrophages affect the tumor microenvironment through a variety of secreted factors and signaling pathways that enhance tumor cell survival in the liver. These signaling pathways work synergistically to regulate the immunosuppressive function of macrophages in the liver, creating a favorable environment for cancer cell dissemination by suppressing immune responses, promoting angiogenesis, and modulating the tumor microenvironment (**Tables 2**, **3**).

**Table 2:** Signaling pathways associated with macrophages in liver metastasis.

Mechanism	Substrate/Target	Pathway	Reference
PI3K-AKT	IGF, miR-106b	Promote cancer invasion, stemness and EMT.	(125, 126)
TGF-P	TGF-6, Smad2/3/4	Promote tumor cells to undergo EMT, guiding the formation of intrahepatic fibrosis pre-metastatic niche (PMN).	(127, 128)
GDNF-GFRA	GDNF, GFRA1	Induce autophagic flux in tumor cells.	(100)
JAK2/STAT3	IL6, FoxQ1	Induce EMT to enhance metastasis mediated by circulating tumor cells.	(129, 130)
EFNB2-EPH B2	EFNB2, EPHB2	Enhance the Warburg effect to promote tumor growth and niche formation.	(131)
CXCL12/CXC R4/NF-KB	CXCL12	Pre-metastatic niche in colorectal liver metastasis (ČRLM).	(90)
CD44/1L-38	GPNMB, IL-33	Promote the expansion and metastasis of cancer stem cells.	(132)
NF-KB	IL-6, IL-8	Enhance the invasive activity of tumor cells.	(133)

**Table 3:** Cytokines related to macrophages in liver metastasis.

Cytokine	Mechanism	Reference
IL-6, HGF, GPNIMB, IGF	Support the expansion of cancer stem cells.	(125, 134)
CCL2, CCL5	Influence the formation of the tumor microenvironment and recruit immune cells to the tumor microenvironment.	(135)
VEGF, IL-1	Promote angiogenesis.	(136)
MMP2	Facilitate the formation of pre-metastatic niches.	(92)
SEMA5A	Enhance the Warburg effect to induce tumor cell proliferationfor metastatic nicheformation.	(137)
IL-1β, IL-6, CCL20, IL-8, TNF-α, CXCL12	Induce EMT in tumor cells.	(61, 78, 98, 138–140)
DOCK7	Regulate tumor cell cholesterol metabolism and increase membrane fluidity.	(106)
IL-10, TGF -β	Inhibit the proliferation and activation of effector T cells, creating an immunosuppressive microenvironment and promoting tumor cell immune escape.	(116, 135)

## 4 Targeting macrophages: a strategy for cancer treatment

### 4.1 Emerging technologies and drugs

As research into the tumor microenvironment (TME) deepens, an increasing number of therapeutic approaches are emerging. A wide variety of emerging technologies and personalized therapies are now on the agenda. Whether targeting macrophage polarization towards the M2 type or addressing macrophage-promoted tumor cell angiogenesis, epithelial-mesenchymal transition (EMT), and immunosuppression for hepatic metastasis, numerous novel approaches have been developed. Targeting the metabolic interactions between tumor cells and tumor-associated macrophages (TAMs) within the TME is a potential strategy for treating tumor cell metastasis. Tumor cell-associated glucose metabolism, such as hypoxia and metabolite secretion, promotes macrophage recruitment and M2-type macrophage polarization. Meanwhile, TAM-related metabolism facilitates tumor cell EMT, invasion, and metastasis (141). Arginine metabolism in the TME also affects TAM differentiation, promoting an increased M2 phenotype. Natural compounds like leucovorin 3’-O-glucoside and indocyanine green, encapsulated in tumor cell-derived particles, target and promote TAM repolarization towards the M1 phenotype (142). A novel class of extracellular vesicle (EV)-like ginseng-derived nanoparticles (GDNPs) isolated from ginseng C. A. Mey plays a role in M1-type and M2-type macrophage polarization. GDNPs can alter M2-type macrophages, inducing M1-type macrophage polarization through Toll-like receptor (TLR)-4 and myeloid differentiation antigen 88 (MyD88)-mediated signaling. GDNPs significantly promote M2 polarization towards the M1 phenotype and generate reactive oxygen species, leading to increased apoptosis in mouse melanoma cells (143). It has been shown that delivering type I interferon (IFNα) to liver metastases via macrophages such as Kupffer cells and tumor-associated macrophages (TAMs) can delay the growth of colorectal and pancreatic ductal adenocarcinoma liver metastases in mice. This may be related to TAM immune activation, enhanced MHC-II-restricted antigen presentation, and reduced depletion of CD8 T cells (120). In one study, it was mentioned that the anti-programmed cell death protein 1 (aPD1) antibody alone had no effect on macrophage recruitment and infiltration into primary colon tumors. However, in tumors treated with regorafenib (REG) and aPD1, there was a significant reduction in intracellularly infiltrated macrophages, and it significantly affected liver metastases. The REG-induced remission of immunosuppression, along with the reactivation of cytotoxic T cells by aPD1, could be related to TAM immune activation, enhanced MHC-II-restricted antigen presentation, and CD8 T cell depletion (120). The reactivation of aPD1-toxic T-cells may explain these occurrences (144). Nanotechnology is now a relatively new field in antitumor research and can solve numerous problems, including the challenge of drug delivery barriers that prevent drugs from accurately reaching their target locations due to multiple barriers within the body. Albumin-conjugated paclitaxel (nAb-PTX) nanoparticles loaded onto nanoporous solid multistage nanovehicles (MSVs) can transport larger amounts to the liver for the treatment of liver metastases. A larger amount of nAb-PTX can be transported to the liver, allowing liver macrophages to retain nAb-PTX, remain viable, and release drugs to inhibit the proliferation of liver metastatic tumor cells and induce apoptosis (145). A novel nanomedicine, composed of KIRA6 (an endoplasmic reticulum stress inhibitor), α-tocopherol nanoemulsion, and anti-PD1 antibody, has been identified. It can transform the hepatic immune microenvironment into a “hot” phenotype by targeting hepatic macrophages to restore the number of CD8^+^ T-cells and promote a “hot” phenotype in immunosuppressed hepatic macrophages. This nanomedicine inhibits tumor cell liver metastasis by targeting hepatic macrophages to restore CD8+ T cell numbers, transforming the hepatic immune microenvironment into a “hot” phenotype, and promoting the repolarization of immunosuppressed hepatic macrophages to restore immune responses (146). Similar results were reported in another study, where TGF β-targeted inhibitory nanomedicines for sonodynamic therapy (SDT) reversed the immunosuppressive tumor microenvironment (147). 25-Hydroxycholesterol (25HC) exhibits a high concentration of aggregates in the TME, and targeting CH25H to disrupt this metabolic checkpoint can manipulate macrophage function to turn cold tumors into hot tumors. This can shape the immunological landscape and overall anti-tumor immune response, offering a promising therapeutic intervention (148). Iron oxide nanoparticles inhibit tumor growth by inducing pro-inflammatory macrophage polarization in tumor tissues (149).In pancreatic cancer, where low drug accumulation rates lead to poor responses to conventional chemotherapy, a technique called ultrasound-targeted microbubble destruction (UTMD) has emerged. UTMD improves chemotherapeutic outcomes by redirecting M2 macrophages to tumor-suppressing M1 macrophages, remodeling vascular normalization, and inducing an anti-tumor immune response, thereby enhancing the chemotherapeutic response (150).

### 4.2 TAM-related pathways in metastasis

As shown in **Table 4**, there are multiple pathways associated with TAMs during tumor cell metastasis.

**Table 4:** TAM-related pathways in metastasis.

Pathway	Substrate/Target	Mechanism	Reference
DUSP2-MAPK/ERK	DUSP2, TGF-β, VEGEA, VEGFD	Promote the M2-like polarization of macrophages, thereby inducing angiogenesis and facilitating the formation of pre-metastatic niches in the liver to accelerate gastric cancer liver metastasis (GOLM).	(105)
PI3K/AKT/mTOR	MiR-4488, RTN3, MMP2	Facilitate the formation of pre-metastatic niches and promote the metastasis of pancreatic neuroendocrine tumors (pNEN).	(92)
mTOR pathway, CTSK/NF- kB	CTSK, IL-10, IL-17	Promote the polarizationof macrophages to the M2 type, where activated M2 macrophages secrete chemokines that enhance the invasion and metastasis of CRC cells.	(151)
PI3K/AKT	ORM1, IL-10	Promote the polarization of macrophages to the M2 type, inducing epithelial-mesenchymal transition (EMT).	(88)
JAK2/STAT3/miR-506 3p/FoxQ1	IL-6, CCL2	Induced EMT and macrophage recruitment.	(129)
TCP4-CCL2-CCR2	TCM4, CCL2	Recruitment of bone marrow-derived TAMs to the metastatic sites in CRC patients.	(51), (152)
GDNF-GFRA1	GDNF	Play a pro-metastatic role by regulating the autophagic flux of tumor cells.	(100)
APOC1- CCL5	CCL5, APOC1	Promote EMT transition to enhance tumor cells metastasis.	(153)

### 4.3 Drugs targeting TAMs to inhibit metastasis

As shown in **Table 5**, drugs targeting TAMs to inhibit tumor cell metastasis.

**Table 5:** Potential drugs targeting TAMs to inhibit metastasis.

Mechanism	Specific Mechanism	Drug	Tumor type	Reference
Deplete TAMS	Inhibi the CSF-1/PI3K/AKT signaling pathway to promote apoptosis of M2 macrophages.	Jiedu Granule formula(JDF)	Hepatocellular carcinoma	(154)
	Activate the JNK1/2 pathway to inhibit macrophage.	Glycyrrhetinic acid	Colorectal cancer	(155)
	M2 polarization Reduce the infiltration of Tregs, naive CD4^+^ T cells and tumor- associated macrophages (TAMs), and reshape the immuno suppressive tumormicroenvironment	Aiduqing formula	Breast cancer	(156)
Induce repolarization of M2-type macrophages	PXB17 repolarizes macrophages to induce CD8 T lymphocyte infiltration in tumors and improve the immunosuppressive microenvironment	CSF1R Inhibitors PXB17	Colorectal cancer	(157)
	Upregulate miR-7083-5p to shift M2 -type TAMs towards an M1-like phenotype, Enhance TAM phagocytosis and cell apoptosis	5-aza-2'-deoxycyti dine (5-aza-dC) and trichostain A (TSA)	Lung cancer	(158)
	Elicited unfolded protein response (UPR), Activated IRE1α to recruit TRAF2, and activated NF-kB to transcribe genes encoding M1 markers in M2 macrophages.	CSF1 Rinhibitors (BLZ945, PLX3397)	Head and neck squamous cell carcinoma	(159)
	Activate the NF-kB-Cyba axis to induce reactive oxygen species (ROS) generation to reprogram TAMS	Carfilzomib	Lung cancer	(160)
	Promote polarization from the M2 to the M1 phenotype and generate total reactive oxygen species.	Vinblasine (VBL)	Colorectal and lung cancer	(161)
	Convert the M2 phenotype to the M1 phenotype of macrophages	ĢDNPs	Melanoma	(143)
	Inhibit CCL2 to suppress TAM recruitment	ginsenoside Rh2	Non- small cell lung cancer	(162)
ReduceTAM	Inhibit the CSF-1/PI3K/AKT signaling pathway to promote apoptosis of M2 macrophages.	CCL2 antibodies CNT0888	Ovarian cancer	(163)

### 4.4 Potential new drugs targeting TAM for the treatment of liver metastasis. Data from clinical trials

Clinical trials of drugs targeting TAMs to inhibit tumor cell metastasis, as shown in **Table 6**.

**Table 6:** Potential new drugs targeting TAM for the treatment of Liver metastasis.

Nct code	Cancer	Drug	Phase	Start date	End date
NCT01437007	Malignant tumors	TKM 080301	Phase 1	2011-08-26	2012-05-21
NCT03274804	colorectal cancer	Pembro lizumaband maaviroc	phase 1	2018-04-01	2020-03-01
NCT05375604	gastric cancer or colorectal cancer	CDK 004	Phase 1	2022-05-28	2023-05-30
NCT05325528	gastric cancer	Tislelizum	Phase 2/3	2022-04-10	2025-12-31 (Estimated)
NCT05877001	colorectal cancer	Tislelizumab	Phase 2	2023-05-30	2025-03-01 (Estimated)
NCT05405685	Pancreatic Cancer	Nimotuzumab and AG	Phase 2	2023-04-27	2025-10-30 (Estimated)
NCT02777710	Pancreatic Cancer and colorectal cancer	Pexidartinib and Durvalumab	Phase 1	2016-05	2019-12
NCT02345408	Pancreatic Cancer	CCX872-B	Phase 1	2015 -02	2020-05-05
NCT01494688	Sold Tumors	ROS5509554	Phase 1	2011-12-20	2018-02-07

Data from ClinicalTrials.

## 5 Traditional Chinese medicine

Chinese medicine possesses the characteristics and advantages of “multi-target, multi-pathway” in tumor treatment. It can exert anti-angiogenic effects by acting on multiple targets, such as HIF-1α, TGF-β1, VEGF, and VEGFR2, and influencing related pathways, including catenin/TCF3/LEF1, mTOR/p70S6K/4E-BP1, Akt/MAPKs/mTOR, p-Src/p-STAT3/VEGF/MMP-2,9, and so on (164). Relevant studies have shown that traditional Chinese medicine (TCM) can regulate the tumor microenvironment (TME) and affect the polarization of M2-type macrophages, thereby inhibiting tumor progression. TCM can exert anti-tumor effects through three pathways: first, by inhibiting macrophage polarization to the M2 phenotype; second, by reversing the M2 type to the M1 macrophage; and third, by inhibiting the role of M2-type macrophages in promoting tumor cell metastasis (165). Dandelion extract mediates the third of these phenotypes by polarizing macrophages from the M2 phenotype to the M1 phenotype (166), and iron in superparamagnetic iron oxide nanoparticles (SPION) achieves a similar effect (167). Macelignan inhibits macrophage M2 polarization via the ROS-mediated PI3K/AKT signaling pathway, thereby blocking IL-1β/NF-κB-dependent CRC metastasis (168). Gambogic acid (GA) inhibits macrophage M2 polarization by reducing tumor cell-derived EV-shuttle miR-21, thereby attenuating CRC liver metastasis (169). Secretion of IL-6 by highly metastatic colon cancer cells induces Kupffer cells to polarize to the M2 type, and KCs M2 polarization is essential for promoting the metastatic ability of colon cancer cells. Toadflax (BU) regulates KCs M2-type polarization and inhibits colon cancer liver metastasis through the SRC-3/IL-6 pathway (170). Astragaloside IV remodels tumor-associated macrophage (TAM) polarization by decreasing M2-type polarization and increasing M1-type polarization, and it inhibits CRC liver metastasis, reduces M2-type macrophage infiltration, and decreases nSMase2 and Rab27a expression in liver metastasis (171). Exosomal CC chemokine ligand-2 (CCL2) promotes the formation of metastatic ecotopes by activating macrophage recruitment and shifting the M1/M2 paradigm to the M2 phenotype. The classic formula in the “Compendium of Golden Chamber Medicinal Formulas” has been used for the treatment of abdominal masses (including tumor diseases), Rhubarb stinging pill, which is mainly used in the clinical treatment of liver fibrosis to reduce hepatic collagen levels, ECM production, and TGF-β1 levels. This formula inhibits the expression of CCL2 in the liver, thereby attenuating macrophage recruitment and polarization to improve the pre-metastatic niche, and then inhibit the liver metastasis of CRC (172). Atractylenolide II (AT-II) inhibits IL-4/IL-13-induced activation of the STAT6 signaling pathway to reduce M2-type macrophage polarization, resulting in fewer lung metastatic nodules (173). Macrophages modified by CAR (CAR-M) have also been initiated for the treatment of solid tumors (174).

## 6 Future perspectives

Currently, using immunotherapy against liver metastases presents a challenge, often associated with macrophage-mediated factors (175). Therapeutic strategies targeting macrophages include tumor-associated macrophage (TAM) depletion, CAR-M series, and M2-type macrophage repolarization (46). For TAM depletion, some studies have suggested using CAR-T cells targeting FRβ+ TAMs to promote their depletion, thereby enhancing endogenous anti-tumor immunity. This offers a promising direction for future immunotherapeutic approaches (176). Regarding macrophage-promoted liver metastasis, with the advent of single-cell sequencing technology and our deeper understanding of TAM subpopulations, we will be able to further explore the relationships between different subpopulations and liver metastasis, facilitating the discovery of new therapeutic targets. In the tumor microenvironment, there is a correlation between TAM heterogeneity and the mechanisms of liver metastasis. Currently, there are limited studies on TAM-related subpopulations and liver metastasis. In the future, we can continue to use single-cell technology and multi-omics analysis to gain a deeper understanding of TAM subpopulations. However, due to the inconsistency in criteria and methods for defining and evaluating TAM heterogeneity, in-depth study of standard TAM subpopulation classification faces challenges. As for current single-cell sequencing, I believe that in the future, SPP1+ TAMs may be an ideal target for adjuvant immunotherapy and improving immunotherapeutic efficacy against C1q+ TAMs, which primarily contribute to the formation of an immunosuppressive microenvironment. However, the intracellular role of C1q+ TAMs within TAMs has not yet been extensively studied. Whether C1q+ TAMs can be used as more specific therapeutic targets in anticancer therapy in the future, or in combination with immune checkpoint inhibitors in cases of resistance, these are promising areas for future exploration. I believe that future research should focus more on how specific subpopulations of tumor-associated macrophages (TAMs) contribute to tumor cell metastasis. Immunotherapy has emerged as a major cancer treatment, but its benefits are limited in patients with liver metastases due to the development of immunotherapeutic resistance. This resistance is often attributed to macrophages, which can affect the efficacy of immune checkpoint blockade. The liver tumor environment is particularly influenced by macrophages, which can impair the recruitment of CD8+ T cells by immunotherapy, leading to immunosuppression in the tumor microenvironment. Therefore, before initiating immunotherapy, it is crucial to identify appropriate treatments to alter the immunosuppressive microenvironment of liver metastases. Research is ongoing to improve drug delivery technologies, such as nanotechnology. The use of nanocarriers may enhance drug accumulation in the liver while minimizing potential negative effects on healthy cells. One study demonstrated that the synergistic treatment of nano-agents with immune checkpoint inhibitors (ICIs) can repolarize macrophages and rejuvenate the liver by remodeling the immunosuppressive microenvironment (146). Another study showed that co-treatment of nano-agents with ICIs could improve the efficacy of immunotherapy by repolarizing macrophages (146). Zhaoting Li et al. (177) utilized a biocompatible alginate-based hydrogel to target Pexidartinib (PLX) to the tumor site and release PLX to block the colony-stimulating factor 1 receptor (CSF1R), thereby depleting TAMs. TAM depletion creates a favorable environment for the local and systemic delivery of anti-programmed cell death protein 1 (aPD-1) antibody-bound platelets to inhibit post-surgical tumor recurrence. The use of nano-agents to induce repolarization of M2-type macrophages improves immunotherapy, offering a better option for overcoming immunotherapy resistance. A lentiviral vector (LV) has also been proposed to deliver interferon alpha (IFNα) to liver metastases via liver macrophages, inhibiting the growth of liver metastases through the activation of antigen presentation and CD8+ T-cell effector function. The use of IFNα in combination with CTLA-4 antibody resolved some resistance mechanisms, such as increased IL-10 signaling, decreased MHC-II restricted antigen presentation in antigen-presenting cells (APCs), and enhanced CTLA-4 expression in T cells (120). In the future, more combination therapies can be explored to target macrophages to release immunosuppression and overcome immunoresistance, including the inhibition of M2-type macrophage polarization and immunotherapy combination therapy. In the tumor microenvironment, TAMs account for a large proportion, and there are other immune cells present, so how to target TAM-mediated liver metastasis is a question to consider. Constructing M2-type macrophage-targeting nanoparticles or using TAM-specific surface markers to design drugs targeting TAM may be viable strategies. With the emergence of new TAM subpopulations identified by single-cell sequencing, this could represent a novel therapeutic approach. It may also be possible to induce the conversion of M2-type macrophages to M1-type by targeting specific signaling pathways associated with the infiltration and polarization of M2-type macrophages, such as the CCL2-CCR2 axis and the STAT3 signaling pathway. Inducing the conversion of M2-type macrophages to M1-type may be a strategy that does not affect other cells, potentially achievable through the use of CpG-targeted liposomes or activation of the TLR signaling pathway. Gene editing techniques may also enable targeting of M2-type macrophages in the future. As new macrophage subpopulations are discovered through single-cell sequencing, drug design studies could involve multiple and more specific targets for drug action. Currently, the identification of macrophage subpopulations needs to be more in-depth to determine the unique functional and phenotypic markers of different subpopulations in order to more accurately target TAMs, which will be more beneficial for highlighting the anticancer therapeutic effects. In the future, TAM subpopulations will become more specific with technologies such as NanoString digital spatial analysis, mass RNA sequencing, or single-cell sequencing, and the newly discovered TAM subpopulations can be used as novel targets. Combination therapies can be developed to inhibit liver metastasis of tumor cells by breaking tumor cell growth or reversing drug resistance. Continued refinement of the delivery system has the potential to improve drug delivery to the tumor microenvironment (TME) and overcome resistance mechanisms. Additionally, emphasis should be placed on the design and rigorous execution of clinical trials, including the promotion of multicenter study designs to improve reliability and the optimization of randomized controlled trial designs to accurately assess the efficacy of targeted macrophage therapy.

## 7 Conclusion

In this paper, it is mentioned that tumor-associated macrophages (TAMs) promote tumor cell growth and liver metastasis through angiogenesis, immunosuppression, and the promotion of epithelial-mesenchymal transition, among other mechanisms. Previously, macrophage classification was limited to the M subpopulations, and research on the influence of the M2 subpopulation on tumor cell development was more restricted. TAMs are heterogeneous, making it challenging to define their subpopulations and functions. However, with the advent of single-cell sequencing technology, macrophage subpopulations have become more specific and detailed. This advancement provides more targets for targeting TAMs to inhibit tumor cell metastasis and offers a deeper understanding of strategies to address resistance to immune checkpoint inhibitors. It enhances the prospects for improving therapeutic efficacy in cancer patients through the in-depth study of macrophage subpopulations and innovative therapeutic approaches. Despite the many hurdles ahead, continued exploration of TAMs is essential. TAMs represent a novel and attractive target that may transform the future landscape of cancer treatment and warrant further research efforts.
